# Postoperative Radiation With or Without Concurrent Chemotherapy for Patients With Locally Advanced Head and Neck Squamous Cell Carcinoma With Multiple Intermediate Risks: A Propensity Score‐Matched Study

**DOI:** 10.1002/cam4.70746

**Published:** 2025-03-07

**Authors:** Thun Leewiboonsilp, Chuleepon Jianpinijnan, Nintita Sripaiboonkij Thokanit, Poompis Pattaranutaporn, Nuttapong Ngamphaiboon

**Affiliations:** ^1^ Division of Medical Oncology, Department of Medicine, Faculty of Medicine, Ramathibodi Hospital Mahidol University Bangkok Thailand; ^2^ Division of Radiation Oncology, Department of Radiology, Faculty of Medicine, Ramathibodi Hospital Mahidol University Bangkok Thailand; ^3^ Ramathibodi Comprehensive Cancer Center, Faculty of Medicine, Ramathibodi Hospital Mahidol University Bangkok Thailand

**Keywords:** adjuvant, chemoradiotherapy, CRT, head and neck squamous cell carcinoma, HNSCC, intermediate risk, locally advanced, postoperative, radiation, radiotherapy

## Abstract

**Background:**

The benefit of postoperative chemoradiotherapy (CRT) over radiotherapy (RT) alone remains unclear for resected locally advanced head and neck squamous cell carcinoma (LA‐HNSCC) patients with intermediate risk(s) such as pT3 or pT4 primary, pN2 or pN3 nodal disease, nodal disease in levels IV or V, perineural invasion (PNI), and lymphovascular invasion (LVI). This study aims to evaluate the benefits of postoperative CRT in patients with multiple intermediate risks.

**Method:**

LA‐HNSCC patients who underwent curative surgery with intermediate risk at our institution were identified. A propensity score‐matched (PSM) method was performed using treatment, sex, age, AJCC staging, LVI, and PNI as covariates.

**Result:**

A total of 162 eligible patients were identified. After PSM, 48 patients were classified into CRT and RT groups, respectively. Baseline characteristics and treatment were well balanced. In the CRT group, most patients received cisplatin‐CRT (67%). Patients with 1, 2, and ≥ 3 intermediate risk factors exhibited significantly different event‐free survival (EFS) (7.2 vs. 3.8 vs. 1.9 years; *p* = 0.007), with a trend towards differences in overall survival (OS) (*p* = 0.068). The median OS of the postoperative RT and CRT groups was not significantly different (4.5 vs. 5.0 years; *p* = 0.950). Similarly, the median EFS was not significantly different (*p* = 0.634). No EFS and OS benefits were demonstrated in patients with ≥ 2 intermediate risk factors treated with CRT compared to RT alone.

**Conclusion:**

In our PSM study, LA‐HNSCC patients with intermediate risk(s) did not benefit from postoperative CRT compared to RT alone. Although patients with multiple intermediate risks had significantly worse survivals, postoperative CRT did not improve OS and EFS. The results of this study may support avoiding unnecessary acute and late toxicity associated with adding chemotherapy to postoperative RT in patients with intermediate risk(s).

## 1 Background

Head and neck squamous cell carcinoma (HNSCC) is a diverse malignancy originating in the oropharynx, lip, oral cavity, larynx, and hypopharynx [1]. Most patients present with locally advanced (LA) disease (stage III or IV), with locoregional recurrence being the most common pattern [2]. Consequently, locoregional treatments such as curative surgery, radiotherapy (RT), or chemoradiotherapy (CRT) are pivotal in managing HNSCC. Surgery is one of the primary approaches for selected resectable and accessible tumors [1]. After curative surgical resection, the EORTC 22931 study demonstrated higher overall survival (OS) and disease‐free survival (DFS) rates in patients treated with postoperative CRT compared to RT alone as an adjuvant setting [3]. Similarly, the RTOG 9501 trial indicated better local and regional control with postoperative treatment, although OS did not significantly differ between the groups [4]. A pooled analysis of RTOG9501 and EORTC 22931 studies, including 793 patients, showed that postoperative CRT improved OS in high‐risk groups with extracapsular extension and positive surgical margins. However, adding cisplatin did not benefit those with intermediate risk factors, such as pathological stage III–IV, lymphovascular invasion (LVI), or perineural invasion (PNI) [5]. Consequently, several guidelines recommend postoperative CRT for high‐risk LA‐HNSCC patients with extracapsular extension and positive surgical margins (R1) [6, 7, 8]. Postoperative RT or CRT improves locoregional recurrence rates and survival benefits in these high‐risk patients [5].

However, adding chemotherapy concurrently with RT for intermediate‐risk LA‐HNSCC patients remains controversial. Many guidelines suggest considering postoperative CRT for these patients but do not provide definitive recommendations on managing single or multiple intermediate risk factors [3, 4]. No prospective clinical trials have evaluated the benefit of postoperative CRT versus RT alone in intermediate‐risk LA‐HNSCC patients [9]. This ambiguity challenges clinicians in selecting appropriate postoperative treatments for intermediate‐risk patients, who may have multiple intermediate risk factors without high‐risk features. Moreover, concurrent chemotherapy with RT significantly increases the risk of acute and late toxicity, such as mucositis, nephrotoxicity, xerostomia, and fibrosis, thereby reducing short‐ and long‐term quality of life [10, 11]. Therefore, identifying predictive factors to better select patients for postoperative CRT or RT alone to maximize clinical benefit and minimize unnecessary side effects is crucial.

Several retrospective studies have evaluated the benefits of postoperative CRT in patients with intermediate risk. A large retrospective study from the US National Cancer Database revealed a survival benefit for postoperative CRT in patients under 70 years old with pT1‐4 and pN2‐3 stages and intermediate risk. However, this benefit may not extend to patients 70 years or older or those with T3‐4N0‐1 disease [12]. Nodal involvement is another factor associated with OS. The survival rate of LA‐HNSCC improved with an increased number of positive lymph nodes when treated with postoperative CRT compared to RT alone [13]. Another retrospective study of 2803 patients with stage III or IV tongue squamous cell carcinoma found that advanced‐stage tongue cancer with two or more metastatic lymph nodes had better survival when receiving postoperative CRT compared to those without lymph node metastasis [14]. However, treatment selection bias due to the retrospective nature of these studies is inevitable, posing challenges in interpreting results and drawing conclusions, as healthier patients are more likely to be offered CRT rather than RT alone. Therefore, our study aims to evaluate the benefits of postoperative CRT compared to RT alone in resected LA‐HNSCC patients with intermediate risk, employing propensity score matching to minimize the treatment selection bias inherent in retrospective studies and balance patient baseline characteristics. In addition, our study evaluated the benefit of postoperative CRT over RT alone for each intermediate risk factor and in patients with multiple intermediate risks.

## 2 Methods

### 2.1 Study Design

This was a retrospective propensity score matched (PSM) study. Data were collected from LA‐HNSCC patients who received definitive surgery, followed by postoperative RT or CRT. Eligible patients treated at our institution between January 2010 and December 2020 were identified through the Ramathibodi cancer registry database. Baseline patient characteristics, pathology results, treatment modalities, and outcomes were collected and entered into our multicenter multidisciplinary electronic database using the RedCap platform [15]. Patients were classified by clinical and pathological risk factors into 3 groups following standard practice guidelines and recommendations: high, intermediate, and low risk [6, 7, 8]. High and low risk groups were excluded from our study. The intermediate risk group was included, and propensity score matching was used to stratify patients into RT and CRT groups with balanced baseline characteristics. After propensity matching, overall survival (OS) and event‐free survival (EFS) were analyzed as primary outcomes. OS was defined as the time from pathological diagnosis to death from any cause. EFS was defined as the time from pathological diagnosis to imaging or pathological confirmation of local progression, distant metastasis, or death from any cause. Local regional free survival (LRFS) was defined as the time from pathological diagnosis to confirmation of local progression or death. Survival status was cross‐checked with the National Security Death Index of Thailand. This study was approved by the Ramathibodi Ethics Committee.

### 2.2 Patient Characteristics

We enrolled patients aged at least 18 years with pathologically confirmed HNSCC (oral cavity, oropharynx, larynx, and hypopharynx) staged I–IVa according to the American Joint Committee on Cancer staging system (AJCC), 8th edition [16]. All eligible patients received definitive treatment with surgery followed by either CRT or RT as curative intent. Patients with intermediate‐risk features (pathological tumor stage III‐IV, positive lymph nodes ≥ 2 or pathological lymph node stage 2–3 (pN2 or pN3), lympho‐vascular invasion (LVI), and perineural invasion (PNI)) were included [5]. The lymph node (LN) risk factor was defined as positive lymph nodes ≥ 2 or pathological lymph node stage II–III. Exclusion criteria were patients who received definitive RT or CRT treatment, patients diagnosed with de novo metastatic disease, and patients with locoregional recurrence who received salvage treatment. High‐risk patients after definitive surgery (margin positive and extracapsular extension of lymph nodes) were excluded, as were patients with nasopharyngeal carcinoma, salivary gland, and paranasal sinus tumors. Unavailable medical records were also excluded. Data collection involved a retrospective review of medical records at Ramathibodi Hospital for baseline characteristics: sex, age, ECOG performance status, smoking status, AJCC 8th edition stage [16], primary tumor site, histological grading, adjuvant RT or CRT, dose and technique of radiation, and dose and regimen of chemotherapy during CRT. Treatment decisions between RT and CRT were made at the physician's discretion and discussed with the patient at the time of treatment.

### 2.3 Statistical Analysis

Baseline patient characteristics and treatment data were analyzed using descriptive statistics. Categorical data were presented as numbers and percentages, with differences compared using Fisher's exact test or Chi‐square test, where appropriate. Continuous data were shown as mean, standard deviation (SD), median, and interquartile range. Propensity score matching (1:1) was used to balance baseline characteristics between RT and CRT groups [17]. To calculate propensity scores, patient age, sex, smoking status, pathological staging, tumor stage, lymph node status, LVI status, and PNI were applied to a logistic regression model for receipt of RT or CRT. OS and EFS were estimated using the Kaplan–Meier method and compared using the propensity score‐weighted log‐rank test. Univariate and multivariate analyses were performed using the Cox proportional hazards model with a significance level of *p* < 0.05. All statistical analyses were conducted using Stata software version 16.0.

## 3 Results

### 3.1 Patient Baseline Characteristics and Treatment

A total of 1305 HNSCC patients were recorded in our multidisciplinary electronic database (Figure S1). Of these, 1019 patients were excluded for not meeting the inclusion criteria. A total of 284 patients were screened and classified into 3 groups: high‐risk (*n* = 73), intermediate‐risk (*n* = 162), and low‐risk (*n* = 49). The 162 intermediate‐risk patients were enrolled in the study and subjected to propensity score matching. After matching using the logistic regression model, the score was computed and divided into 7 groups. The chosen variables included sex, age group, AJCC 8th staging, LVI, and PNI status. After matching, 96 patients were classified into two groups: RT and CRT, with 48 patients in each group (Figures S1, S2 and Table S3). The demographic and disease characteristics are summarized in Table 1 for both pre‐matching and post‐matching groups. In the pre‐matching group, 162 patients were classified into 74 in the CRT group and 88 in the RT group. AJCC 8th stage IV disease and lymph node risk factors were significantly different in the CRT group compared to the RT group (*p* < 0.001). There were no statistically significant differences in other demographic data. After propensity score matching, the baseline characteristics of 48 patients in each of the CRT and RT groups were well balanced. Most patients had pT3‐4 (80%), pN2‐3 or positive lymph nodes ≥ 2 (80%). LVI and PNI positive status were 31% and 29%, respectively. No patients with pN3 were included in our study. Cisplatin was the most common regimen (67%) in the CRT group, while weekly carboplatin was used for cisplatin‐ineligible patients (33%). The cumulative cisplatin dose during CRT was 223 (± 57) mg/^2^m^2^. For radiation techniques, Intensity Modulated Radiation Therapy (IMRT)/Volumetric Modulated Arc Therapy (VMAT) was used in 44 patients (45%), and the 3D technique was used in 39 patients (40%). Both techniques were the main approaches, with a mean cumulative radiation dose of 61–64 Gy. Thirteen patients (15%) were treated with the 2D technique with a mean cumulative radiation dose of 60 ± 5.3 Gy.

**TABLE 1:** Baseline patient characteristics and postoperative treatment of LA‐HNSCC patients with intermediate risk factor(s): Pre‐ and post‐propensity score matching.

Characteristics	Before matching			After matching		
	CRT *N* = 74 (%)	RT *N* = 88 (%)	*p*	CRT *N* = 48 (%)	RT *N* = 48 (%)	*p*
Median age (range)	57 (20–85)	62 (21–94)		58 (20–85)	61 (28–85)	
≥ 65	15 (20)	33 (37)	0.017	15 (31)	18 (38)	0.519
Gender						
Male	56 (76)	64 (73)	0.670	31 (65)	34 (71)	0.513
Female	18 (24)	24 (27)		17 (35)	14 (29)	
ECOG performance status						
0–1	68 (92)	82 (93)	0.755	45 (94)	44 (92)	0.695
2	6 (8)	6 (7)		3 (6)	4 (8)	
Smoking status						
Never	36 (49)	40 (45)	0.685	28 (58)	21 (44)	0.153
Ever	38 (51)	48 (55)		20 (42)	27 (56)	
Mean pack year(±SD)	18 (±27.4)	13 (±19.3)		12 (±18.9)	19 (±26.1)	
Primary tumor site						
Oral cavity	48 (65)	55 (63)	0.356	33 (69)	33 (63)	0.268
Oropharynx	7 (10)	5 (6)		4 (8)	2 (4)	
Larynx	15 (20)	26 (29)		8 (17)	15 (31)	
Hypopharynx	4 (5)	2 (2)		3 (6)	1 (2)	
The AJCC 8th staging						
I‐II	1 (1)	11 (13)	< 0.001	1 (2)	1 (2)	0.572
III	13 (19)	37 (42)		13 (27)	13 (27)	
IVa	60 (81)	40 (45)		34 (70)	34 (70)	
Tumor stage						
pT1‐2	13 (18)	22 (25)	0.252	9 (19)	10 (21)	0.798
pT3‐4	61 (82)	66 (75)		39 (81)	38 (79)	
Lymph node risk—Positive	37 (50)	18 (20)	< 0.001	22 (46)	15 (31)	0.142
LVI—Positive	33 (45)	27 (31)	0.068	13 (27)	17 (35)	0.378
PNI—Positive	25 (34)	33 (38)	0.623	13 (27)	15 (31)	0.653
Summary of risk factor						
1 factor	24 (32)	45 (51)	0.016	20 (42)	21 (44)	0.837
≥ 2 factors	50 (68)	43 (49)		28 (58)	27 (56)	
Radiotherapy technique						
2D	9 (12)	13 (15)	0.705	7 (15)	6 (12)	0.490
3D	32 (43)	39 (44)		18 (37)	21 (44)	
IMRT/VMAT	33 (45)	36 (41)		23 (48)	21 (44)	
Mean actual RT dose (Gy) (±SD)	64 (±4.2)	61 (±3.5)		64 (±3.8)	61 (±4.4)	

### 3.2 Survival Outcomes of Each and Multiple Intermediate Risk Factors

**FIGURE 1:**
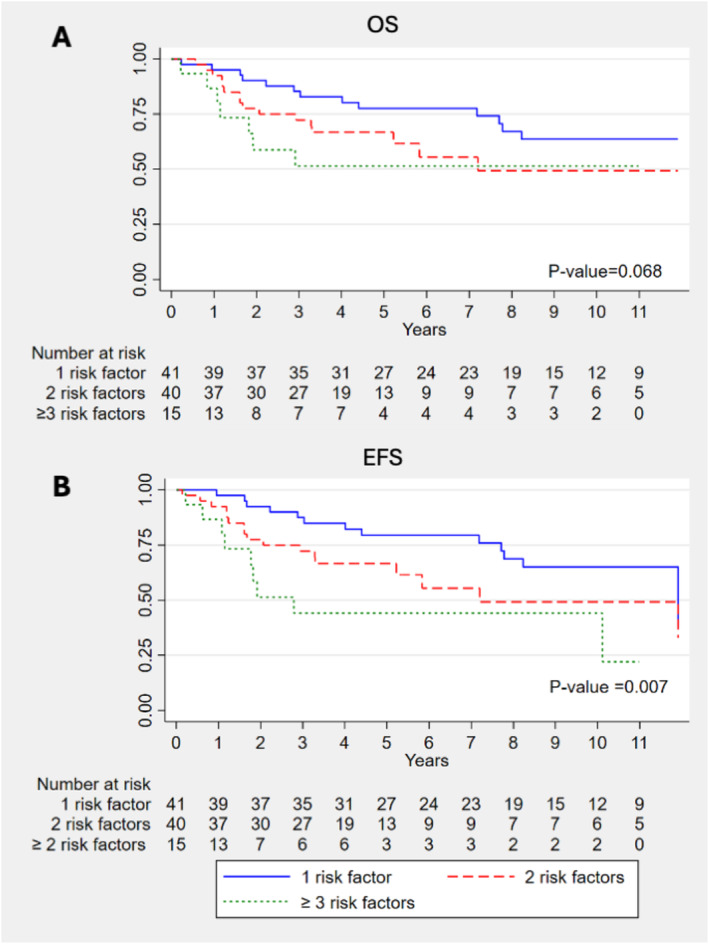
OS and EFS of summary of intermediate risk factors.

The median follow‐up duration of the study was 4.68 years. OS and EFS for each intermediate risk factor, regardless of postoperative treatment, are shown in Figures S4–S11. Patients with positive LVI had significantly worse OS (6.1 vs. 3.1 years; *p* = 0.004) and EFS (6.1 vs. 2.9 years; *p* < 0.001) (Figures S8 and S9). Additionally, patients with 1, 2, and ≥ 3 intermediate risk factors exhibited significantly different EFS (7.2 vs. 3.8 vs. 1.9 years; *p* = 0.007), with a trend towards differences in OS (7.4 vs. 3.6 vs. 2.3 years; *p* = 0.068) (Figure 1). There was no significant difference in OS and EFS between patients who received postoperative CRT with cisplatin or carboplatin (Figures S12 and S13).

### 3.3 Survival Benefits of Postoperative RT vs. CRT in Patients With Intermediate Risk Factor(s)

**FIGURE 2:**
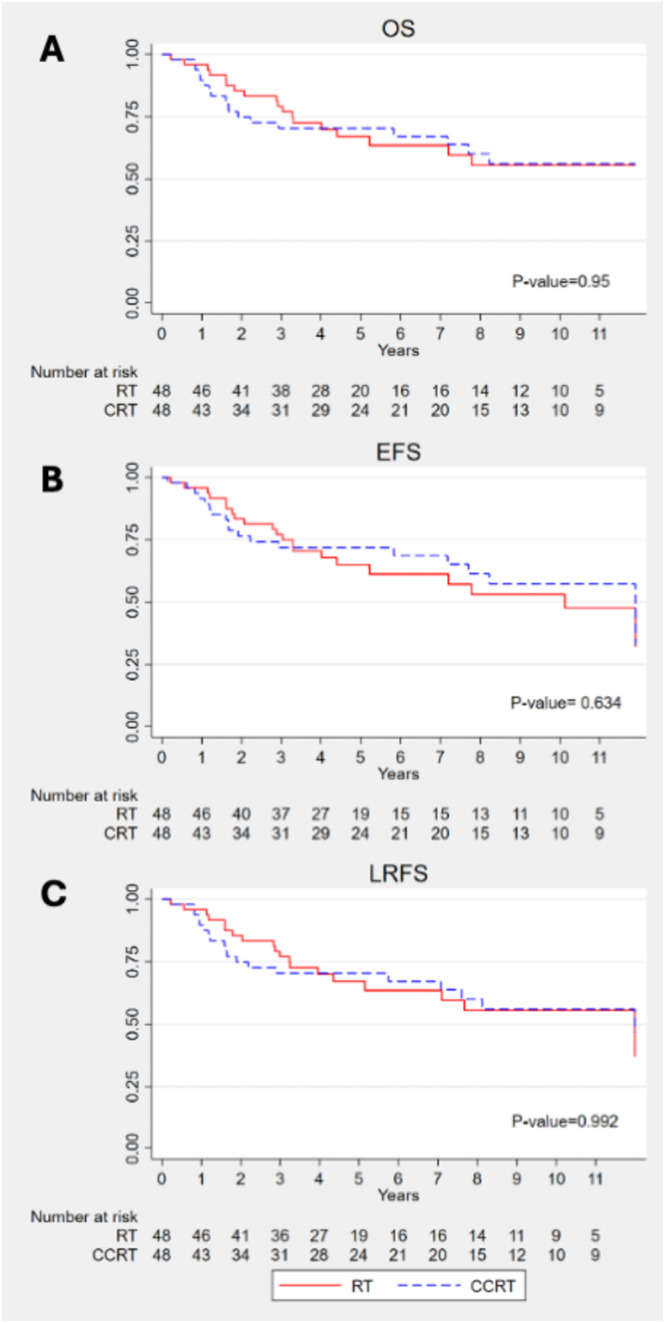
OS, EFS, and LRFS of LA‐HNSCC patients with intermediate risk factors treated with postoperative RT or CRT.

The median OS of the postoperative RT and CRT groups in LA‐HNSCC patients was not significantly different (4.5 vs. 5.0 years; *p* = 0.950) (Figure 2). The 5‐year OS rates were 67.1% for the RT group and 70.3% for the CRT group. Similarly, the median EFS in the RT and CRT groups was not significantly different (4.3 vs. 5.0 years; *p* = 0.634), with 5‐year EFS rates of 64.9% and 71.8%, respectively. The median LRFS was 4.5 years in the RT group and 4.9 years in the CRT group (*p* = 0.992).

**FIGURE 3:**
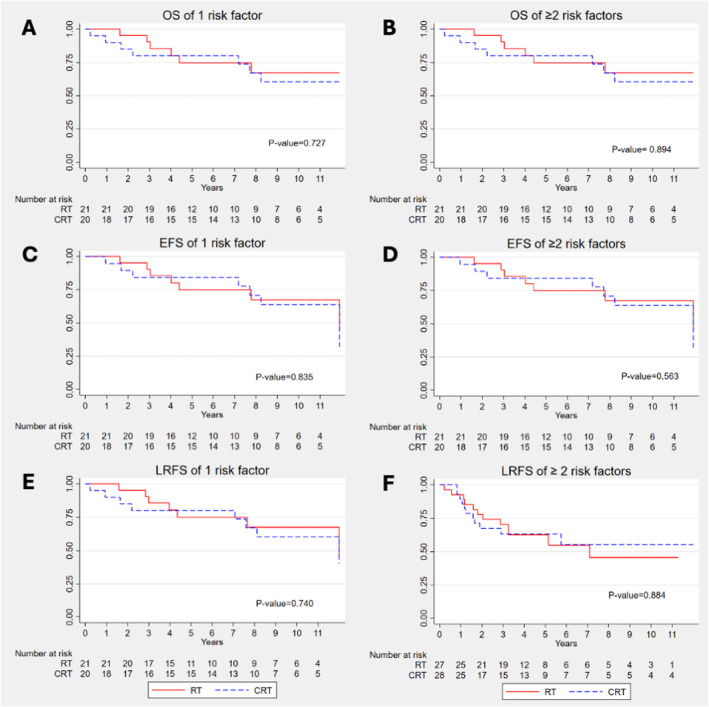
OS, EFS, and LRFS of LA‐HNSCC patients with single and multiple (≥ 2) intermediate risk factors treated with postoperative RT or CRT.

There was no statistically significant difference in survival for patients with single or multiple intermediate risk factors who received postoperative CRT or RT alone (Figure 3). In the single risk factor group, the 5‐year OS rates for patients treated with RT alone and CRT were 74% and 80%, respectively (*p* = 0.727). For patients with multiple intermediate risk factors, the 5‐year OS rates were 62% for the RT group and 63% for the CRT group (*p* = 0.894).

**FIGURE 4:**
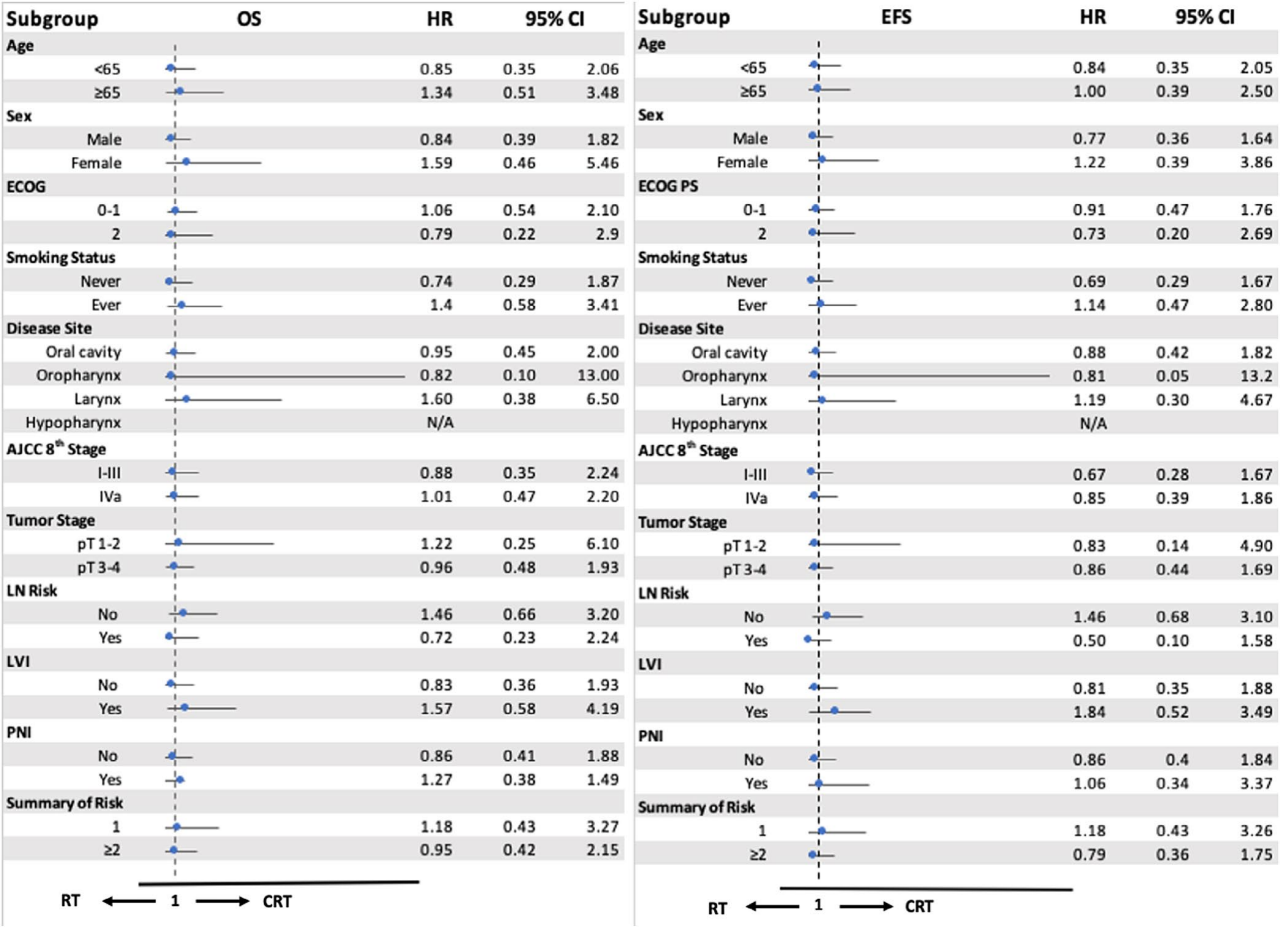
Subset analysis of LA‐HNSCC patients with intermediate risk factors treated with postoperative RT or CRT.

In the univariate and multivariate analyses, only patients aged ≥ 65 years and those with positive LVI were significantly associated with worse EFS and OS (Table 2). Other intermediate risk factors, such as pathological tumor stage III–IV, LN risk, PNI status, and multiple intermediate risk factors, were not significantly associated with survivals. In the subgroup analysis for both OS and EFS, there were no significant differences between RT and CRT in each subgroup, such as age ≥ 65, AJCC stage IVa, and positive LVI (Figure 4).

**TABLE 2:** Univariate and multivariate analysis for EFS and OS of LA‐HNSCC patients with intermediate risk factor(s).

Factors	EFS				OS			
	Univariate		Multivariate		Univariate		Multivariate	
	HR (95% CI)	*p*	HR (95% CI)	*p*	HR (95% CI)	*p*	HR (95% CI)	*p*
Gender Female vs. male (ref)	0.75 (0.38–1.47)	0.401			0.75 (0.37–1.50)	0.407		
Age group ≥ 65 vs. < 65 (ref)	2.27 (1.22–4.21)	0.009	2.13 (1.14–3.98)	0.010	2.00 (1.06–3.80)	0.034	1.88 (0.98–3.59)	0.056
Smoking Ever vs. never (ref)	1.11 (0.59–2.06)	0.739			1.20 (0.64–2.29)	0.565		
ECOG 2 vs. 0–1 (ref)	1.13 (0.39–3.22)	0.826			1.38 (0.48–3.94)	0.548		
AJCC 8th staging IV vs. I‐III (ref)	0.72 (0.38–1.39)	0.337			0.86 (0.43–1.71)	0.661		
pT stage pT3‐4 vs. pT1‐2 (ref)	1.92 (0.75–4.90)	0.171			1.38 (0.58–3.31)	0.469		
LN risk Yes vs. no (ref)	0.6 (0.30–1.19)	0.149			0.65 (0.32–1.3)	0.222		
LVI Yes vs. no (ref)	3.55 (1.83–6.90)	< 0.001	3.41 (1.74–6.69)	< 0.001	2.68 (1.37–5.23)	0.002	2.59 (1.31–5.12)	0.006
PNI Yes vs. no (ref)	1.77 (0.90–3.50)	0.099			1.36 (0.67–2.77)	0.400		
Sum of risk factor ≥ 2 vs. 1 (ref)	1.84 (0.97–3.52)	0.064			1.57 (0.81–3.02)	0.181		
Treatment group CRT vs. RT (ref)	0.86 (0.46–1.61)	0.634	1.05 (0.55–2.01)	0.160	0.98 (0.52–1.86)	0.950	1.19 (0.62–2.20)	0.611

## 4 Discussion

In our propensity scored matched study, OS, EFS, and LRFS among resected LA‐HNSCC patients with intermediate risk factors showed no significant differences between the RT and CRT groups, including patients with multiple intermediate risk factors. Subgroup analyses did not reveal any specific patient subgroup that benefited from postoperative CRT. These findings align with previous studies reported in the literature. In the RTOG 9501 and EORTC 22931 studies, patients with intermediate risk factors showed no difference in OS and DFS between CRT and RT in pooled analyses [3, 4, 5]. Similarly, a retrospective PSM study of LA‐HNSCC patients with intermediate risk found no survival benefit or improvement in locoregional control for patients who received postoperative CRT compared to RT alone [18]. However, a subset analysis of this study showed improved locoregional relapse‐free survival in intermediate‐risk patients with pT3–4 or pN2–3 who were treated with CRT rather than RT alone, which differed from our results. A potential explanation for this discrepancy could be that our study did not include patients with the most advanced locoregional disease, particularly those classified as pN3. Moreover, two large retrospective studies using the US National Cancer Database to assess outcomes of postoperative RT vs. CRT in LA‐HNSCC patients with intermediate risk found that CRT was associated with a statistically significant improvement in OS compared to RT alone. In these studies, the survival benefits of CRT over RT alone were more pronounced but remained limited to patients with multiple positive LNs and those under 70 years old with non‐oropharyngeal pT1‐4 N2‐3 disease [12, 13]. While these studies suggest a benefit from postoperative CRT, the inevitable treatment selection bias due to the retrospective nature of these studies poses significant challenges in interpreting results and drawing conclusions. Recently, preliminary results from a randomized controlled phase 3 study comparing postoperative RT with or without cetuximab in patients with intermediate risk have shown an improvement in disease‐free survival (DFS) favoring the cetuximab –RT combination [19]. However, the benefit to OS remains inconclusive at this preliminary result. Given that several phase 3 randomized studies have consistently shown that cetuximab–RT is inferior to cisplatin–CRT in a definitive setting, it is crucial to follow the final report of this study for a comprehensive evaluation [20, 21, 22].

In our study, multiple intermediate risk factors were significantly associated with poorer survival outcomes, consistent with a previous retrospective study [23]. In contrast, a retrospective study of resected oral cavity squamous cell carcinoma (OCSCC) patients with 3 intermediate risk factors who received postoperative CRT demonstrated significant survival benefits compared to RT alone [24]. However, this study included only OCSCC patients and used a non‐propensity score matched retrospective design.

Our retrospective study was strengthened by using a propensity score matching method to stratify risk factors. All baseline characteristics and treatments were well balanced, which may minimize selection bias. However, the propensity score matching method led to significant patient loss, resulting in a relatively small sample size for analysis. Additionally, the inherent limitations of a retrospective study remain. All treatment decisions depended on each physician and patient performance status, with various follow‐up durations. Although primary tumors of the oropharynx and oral cavity with level III or IV cervical nodes are considered intermediate risk factors based on previous meta‐analysis [5], we did not include these in our study due to the lack of specific lymph node level classification in the database. Although cisplatin was used in the majority of CRT patients, a small number of cisplatin‐ineligible patients in our study received carboplatin concurrently with postoperative RT. As there are limited data on postoperative systemic treatment regimens for CRT, carboplatin is frequently used for cisplatin‐ineligible HNSCC patients in Thailand, as cetuximab is currently not reimbursable [25]. In this study, we did not observe differences in the survival of intermediate risk patients treated with postoperative cisplatin vs. carboplatin CRT. A randomized controlled study is warranted to evaluate the benefit of postoperative CRT in resected LA‐HNSCC patients with intermediate risk, especially in cisplatin‐ineligible patients.

Based on the current evidence, the unclear benefit of postoperative CRT over RT alone in LA‐HNSCC patients with intermediate risk remains. It might continue to be challenging for physicians to recommend adding cisplatin to postoperative RT for LA‐HNSCC patients, especially those with intermediate risk(s). The results of this study may support avoiding unnecessary acute and late toxicity associated with adding chemotherapy to postoperative RT in patients with intermediate risk factors [10, 11]. Minimal residual disease (MRD) testing may play a future role in guiding physicians to select the proper patients with intermediate risk who may benefit from CRT over RT alone. Circulating tumor DNA (ctDNA) has been evaluated in LA‐HNSCC patients who received both definitive and adjuvant treatment. A study using a tumor‐agnostic plasma ctDNA assay with a 26‐gene next‐generation sequencing panel to evaluate the presence of MRD after curative treatment of LA‐HNSCC patients demonstrated a significantly higher 2‐year PFS rate in MRD‐negative patients [26]. Additionally, a plasma extracellular vesicle microRNA model was developed in LA‐HNSCC patients to evaluate MRD post‐definitive treatment [27]. The dynamic changes of miRNA‐491‐5p pre‐ and post‐curative treatment were significantly associated with DFS and OS. These MRD tests may play a future role in assisting physicians to select proper postoperative treatments to maximize benefits and minimize toxicity for resected LA‐HNSCC patients.

## 5 Conclusion

In our propensity score matching study, resected LA‐HNSCC patients with intermediate risk(s) did not benefit from postoperative CRT compared to RT alone. Although patients with multiple intermediate risk factors had significantly worse survival outcomes, postoperative CRT did not improve OS and EFS in this group. The results of this study may support avoiding unnecessary acute and late toxicity associated with adding chemotherapy to postoperative RT in patients with intermediate risk factors. Further prospective or randomized controlled studies are warranted to evaluate the benefit of postoperative CRT in resected LA‐HNSCC patients with intermediate risk factors.

## Author Contributions

**Thun Leewiboonsilp:** conceptualization (supporting), data curation (lead), formal analysis (lead), methodology (supporting), writing – original draft (lead). **Chuleepon Jianpinijnan:** data curation (supporting), writing – review and editing (supporting). **Nintita Sripaiboonkij Thokanit:** formal analysis (supporting), software (equal), validation (supporting). **Poompis Pattaranutaporn:** software (equal), visualization (supporting). **Nuttapong Ngamphaiboon:** conceptualization (lead), funding acquisition (lead), investigation (lead), methodology (lead), project administration (lead), resources (lead), supervision (lead), visualization (lead), writing – review and editing (lead).

## Ethics Statement

This study was approved by the Ramathibodi Ethics Committee of Ramathibodi Hospital, Mahidol University, Bangkok, Thailand. A waiver of written informed consent was granted by the Ethics Committee due to the retrospective nature of the study, which involved the use of de‐identified patient data. The study was conducted in accordance with the ethical standards of our institution and in compliance with the Declaration of Helsinki.

## Conflicts of Interest

N. Ngamphaiboon reports institutional research funding from MSD, Roche, RAPT therapeutics, BeiGene, AstraZeneca, Merus, and Boehringer Ingelheim Pharmaceuticals; and personal fees and nonfinancial support from MSD, Roche, Merck, Eisai, BMS, BeiGene, Alliance Pharma, Ipsen, Servier, and Ascendant Biotech Corporation. The other authors declare no Conflicts of Interest.

## Declaration of Generative Ai and Ai‐Assisted Technologies in the Writing Process

During the preparation of this work, the author used ChatGPT (GPT‐ 4o) solely to enhance readability and correct grammar. ChatGPT was not involved in any other conceptual framework aspects of the work, such as study design, data analysis, interpretation, reference sourcing, or manuscript drafting. After using ChatGPT, the authors reviewed and edited the content as needed and take full responsibility for the content of the publication.

## Presentation

The abstract of the study was accepted for the Mini‐oral presentation at the 22nd Annual Meeting of the Japanese Society of Medical Oncology (JSMO2025), March 6, 2025, Kobe, Japan.

## Supporting information


Data S1.

## Data Availability

The data that support the findings of this study are available from the corresponding author upon reasonable request.
