# Supplementary material for: Postoperative Radiation With or Without Concurrent Chemotherapy for Patients With Locally Advanced Head and Neck Squamous Cell Carcinoma With Multiple Intermediate Risks: A Propensity Score‐Matched Study
Source: Cancer Med. 2025 Mar 7;14(5):e70746. doi: 10.1002/cam4.70746 (PMC11886883; doi:10.1002/cam4.70746)
Supplement: Supplementary file 1 — Data S1. [file CAM4-14-e70746-s001.docx]

**Supplement 1.** Consort diagram

Head and neck patient in Ramathibodi multidisciplinary database since 2010-2021
(N=1305)

1019 patients were excluded due to not meet to inclusion criteria

284 patients were included

Patients were classified into risk groups after definitive surgery

122 patients were excluded

- 73 patients with high risk feature

- 49 patients with low risk feature

CRT group (N=48)

162 patients with intermediate risk groups were included. The propensity score matching was analyzed and classified by treatment group

66 patients were excluded for propensity score matching

RT group (N=48)

**Supplement 2.** Propensity score matching presented in histogram

**
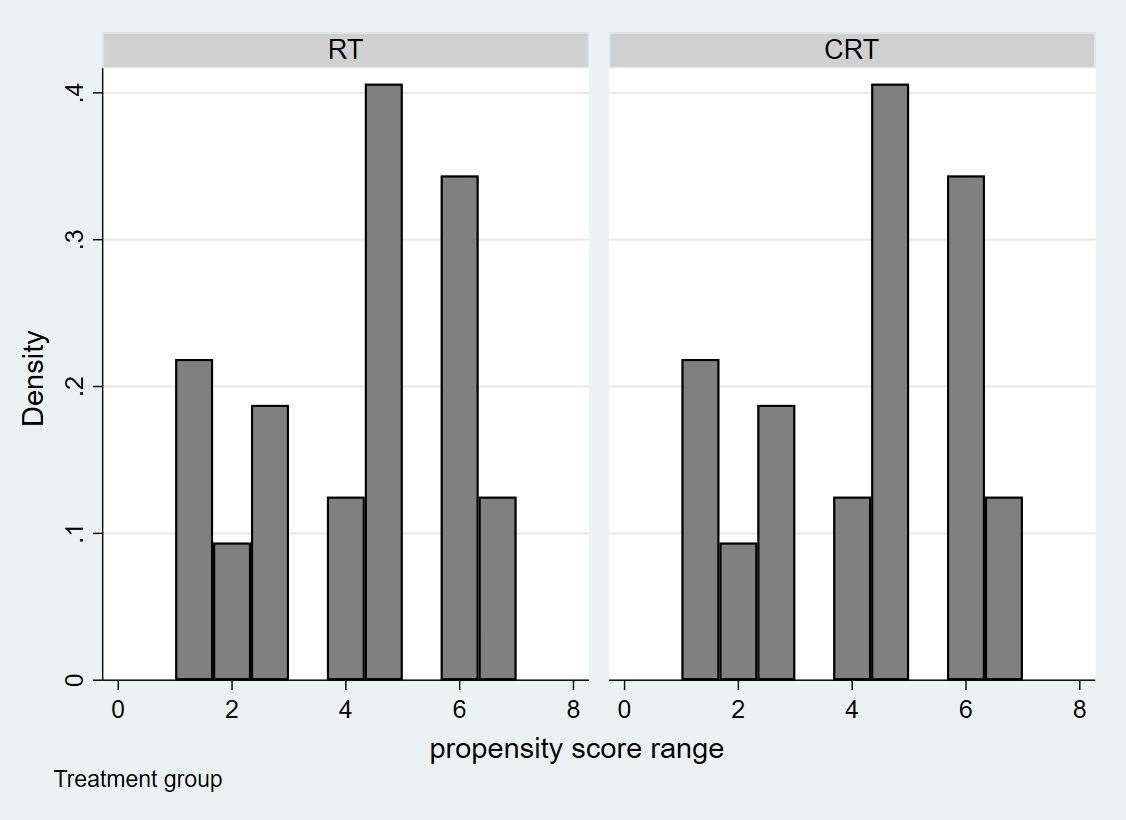
**

**Supplement 3.** Distribution of select covariate by propensity score declines

|  | **Range of Propensity Score** | | | | | | |
| --- | --- | --- | --- | --- | --- | --- | --- |
|  | 1 (0.1-0.19) | 2 (0.2-0.29) | 3 (0.3-0.39) | 4 (0.4-0.49) | 5 (0.5-0.59) | 6 (0.6-0.69) | 7 (0.7-0.79) |
| **Treatment group** |  |  |  |  |  |  |  |
| CRT | 7 | 3 | 6 | 4 | 13 | 11 | 4 |
| RT | 7 | 3 | 6 | 4 | 13 | 11 | 4 |
| **Sex** |  |  |  |  |  |  |  |
| Male | 9 | 5 | 8 | 7 | 11 | 19 | 6 |
| Female | 5 | 1 | 4 | 1 | 15 | 3 | 2 |
| **Mean age, years** |  |  |  |  |  |  |  |
| CRT | 69.5 | 50.6 | 50.7 | 67.9 | 53.1 | 56.5 | 62 |
| RT | 74.1 | 62.8 | 62 | 78.5 | 59 | 56.3 | 58.9 |
| **The AJCC 8^th^ stage** |  |  |  |  |  |  |  |
| stage I-III | 3 | 2 | 2 | 3 | 4 | 4 | 1 |
| stage IVa | 11 | 4 | 10 | 5 | 22 | 18 | 7 |
| **LVI** |  |  |  |  |  |  |  |
| Negative | 11 | 5 | 4 | 8 | 19 | 19 | 0 |
| Positive | 3 | 1 | 8 | 0 | 7 | 3 | 8 |
| **PNI** |  |  |  |  |  |  |  |
| Negative | 8 | 5 | 6 | 8 | 17 | 19 | 5 |
| Positive | 6 | 1 | 6 | 0 | 9 | 3 | 3 |

**Supplement 4.** OS by the 8^th^ AJCC pathological tumor stage

**
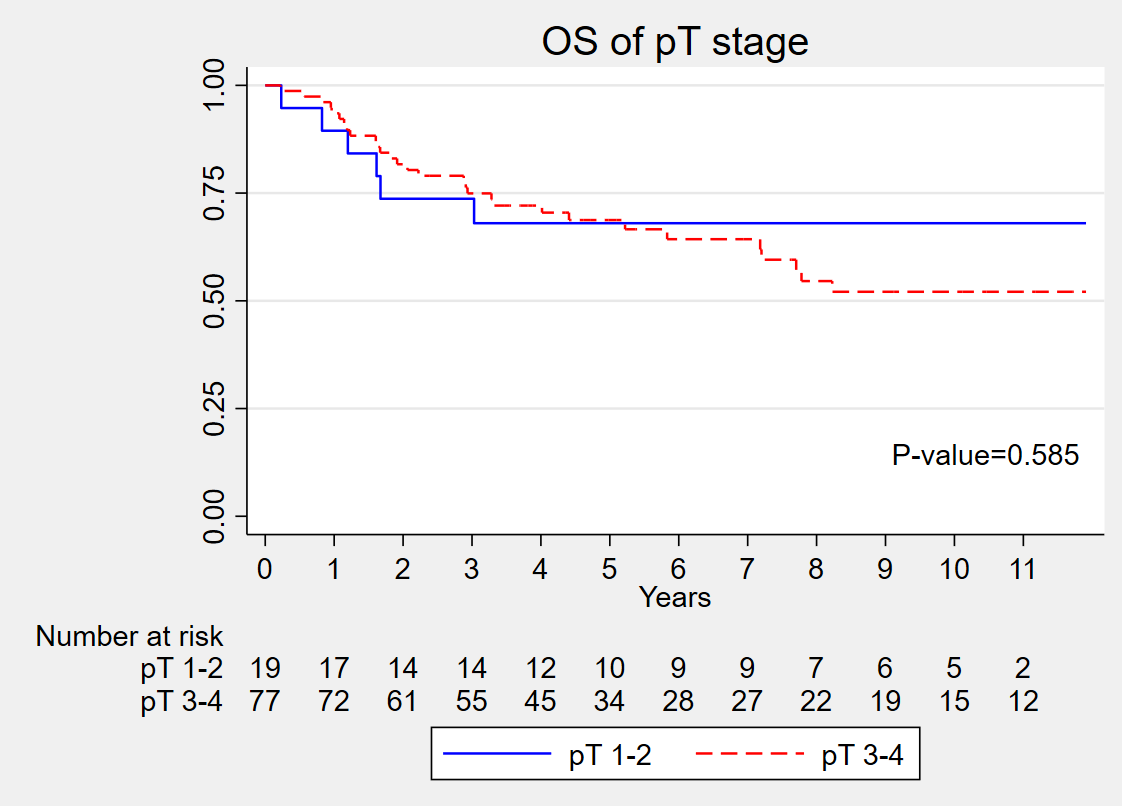
**

HR 1.28 (95%CI 0.53-3.07); p-value =0.585

Median OS (range) years:

-pT stage I-II: 5 (0.2-15) years

-pT stage III-IV: 4.6 (0.2-15) years

**Supplement 5.** EFS by the 8^th^ AJCC pathological tumor stage


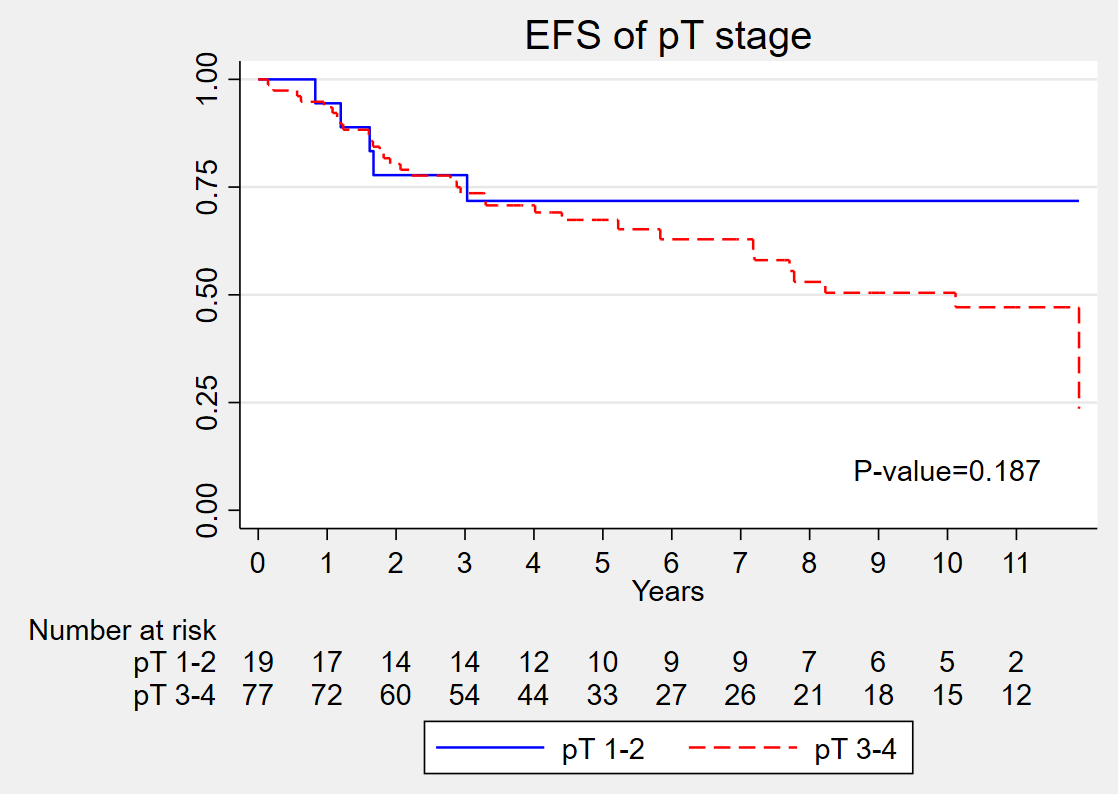


HR 1.88 (95%CI 0.74-4.79); p-value =0.187

Median EFS (range) years:

- pT stage I-II: 5 (0.2-15) years

- pT stage III-IV: 4.4 (0.1-15) years

**Supplement 6.** OS by lymph node risk factor (classified as pathologic lymph node 2-3 and/or number of lymph node positive ≥2)

**
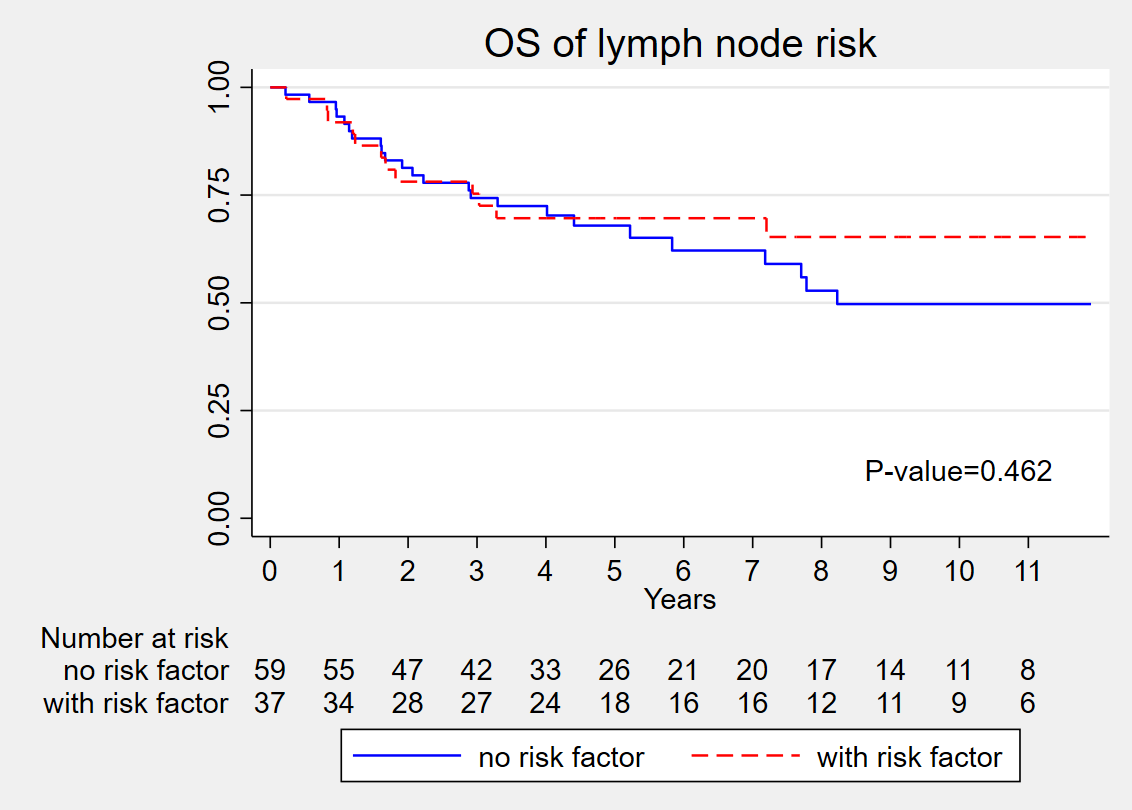
**

HR 0.77 (95%CI 0.39-1.54); p-value =0.462

Median OS (range) years:

- No LN risk: 4.4 (0.2-13.6) years

- With LN risk: 4.9 (0.2-13.8) years

**Supplement 7.** EFS by lymph node risk factor (classified as pathologic lymph node 2-3 and/or number of lymph node positive ≥2)


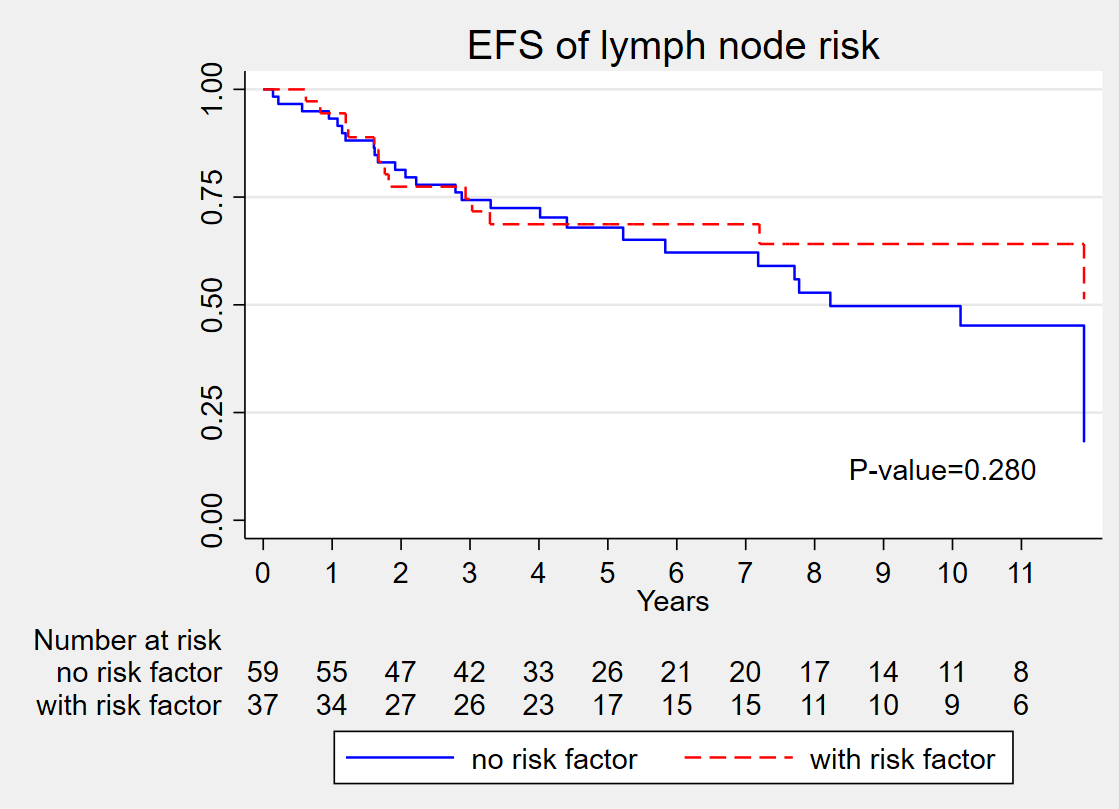


HR 0.70 (95%CI 0.36-1.34); p-value =0.280

Median EFS (range) years:

- No LN risk: 4.4 (0.1-13.6) years

- With LN risk: 4.7 (0.2-15.2) years

**Supplement 8.** OS by lympho-vascular invasion (LVI) status


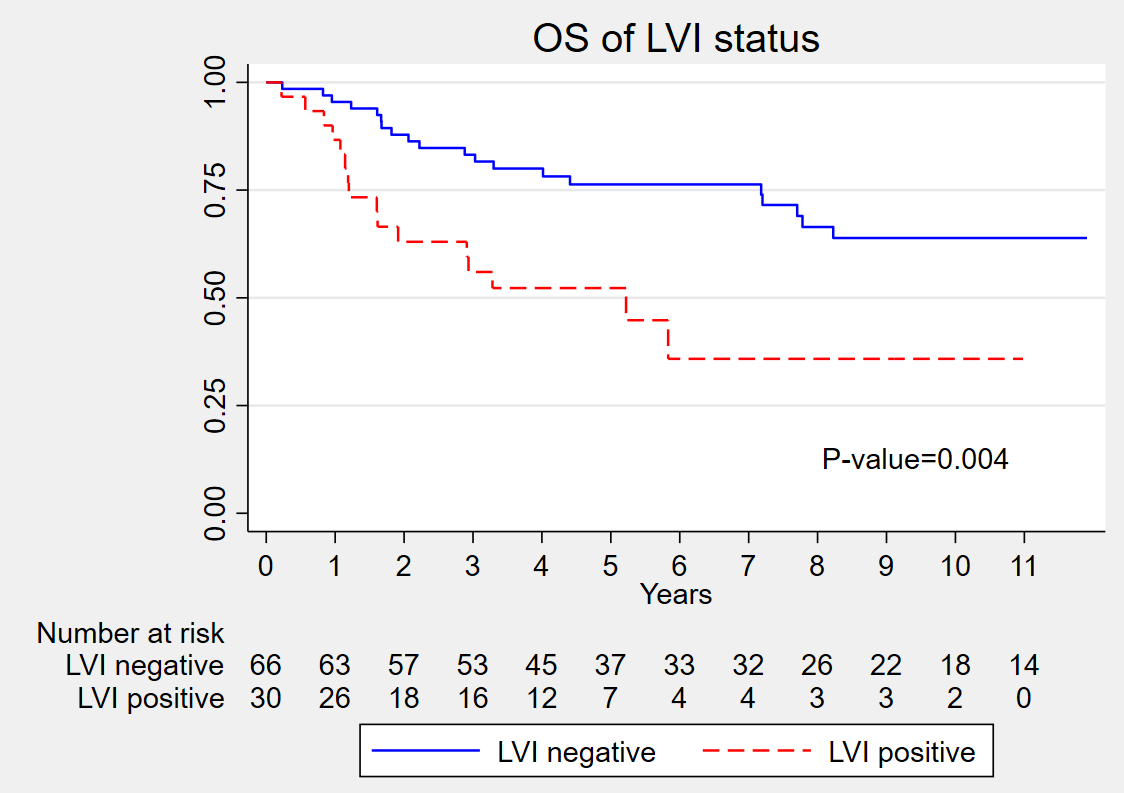


HR 2.68 (95%CI 1.37-5.23); p-value =0.004

Median OS (range) years:

- LVI negative: 6.1 (0.2-15.3) years

- LVI positive: 3.1 (0.2-10.9) years

**Supplement 9.** EFS by lympho-vascular invasion (LVI) status


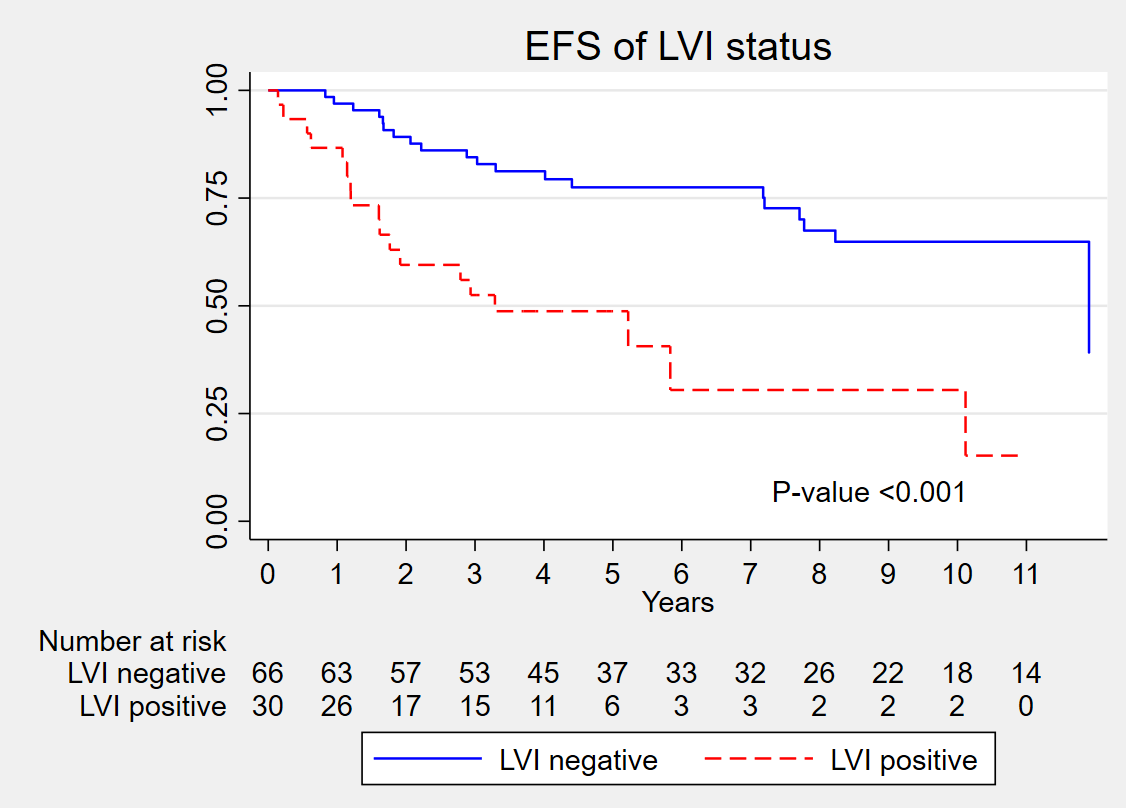


HR 3.56 (95%CI 1.83-6.90); p-value <0.001

Median EFS (range) years:

- LVI negative: 6.1 (0.2-15.3) years

- LVI positive: 2.9 (0.1-10.4) years

**Supplement 10.** OS by perineural invasion (PNI) status


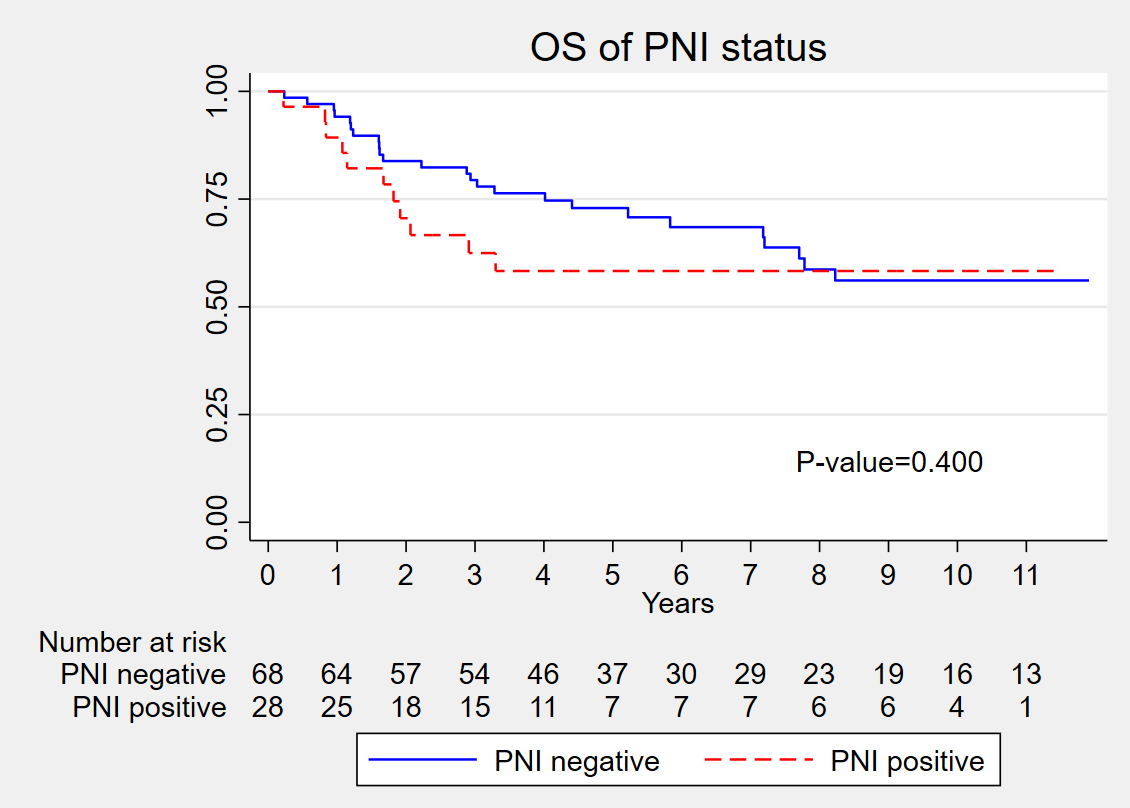


HR 1.36 (95%CI 0.67-2.77); p-value = 0.400

Median OS (range) years:

- PNI negative: 4.2 (0.2-15.3) years

- PNI positive: 3.5 (0.1-11.4) years

**Supplement 11.** EFS by perineural invasion (PNI) status


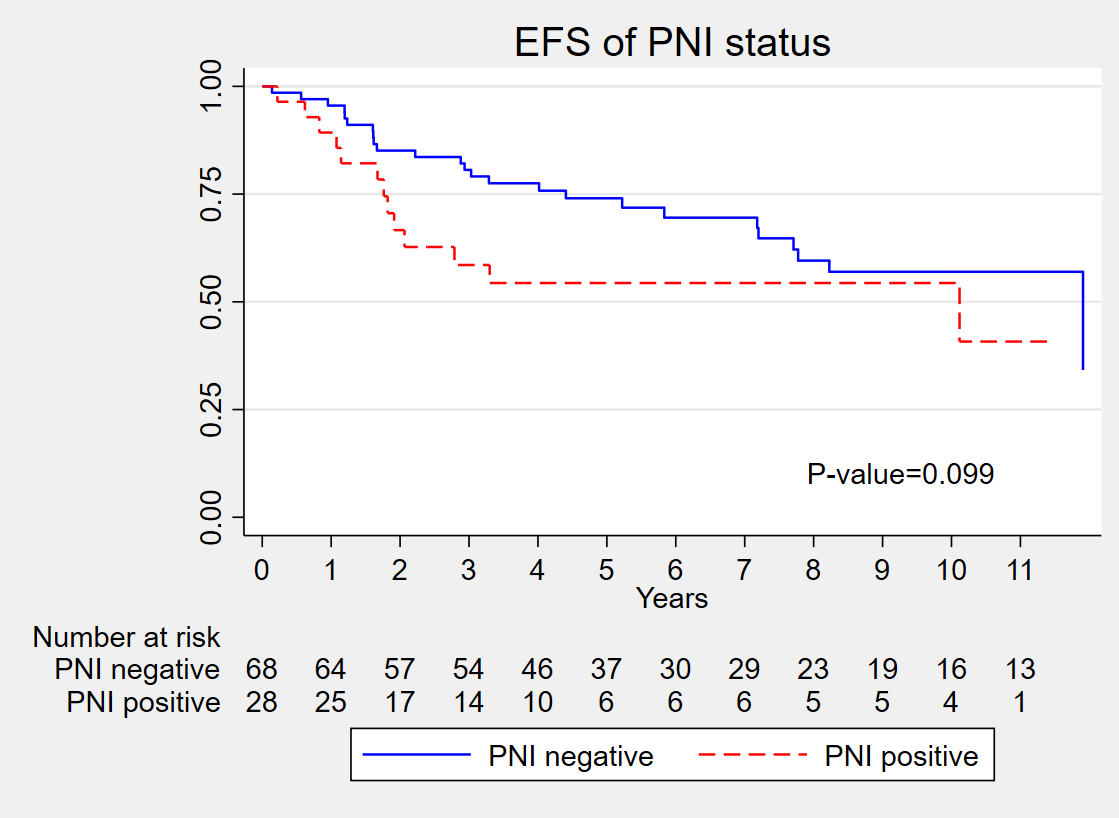


HR 1.77 (95%CI 0.89-3.50); p-value =0.099

Median EFS (range) years:

- PNI negative: 5.1(0.2-15.3) years

- PNI positive: 3(0.2-11.4) years

**Supplement 12.** OS by chemotherapy regimen during chemoradiotherapy and radiotherapy alone


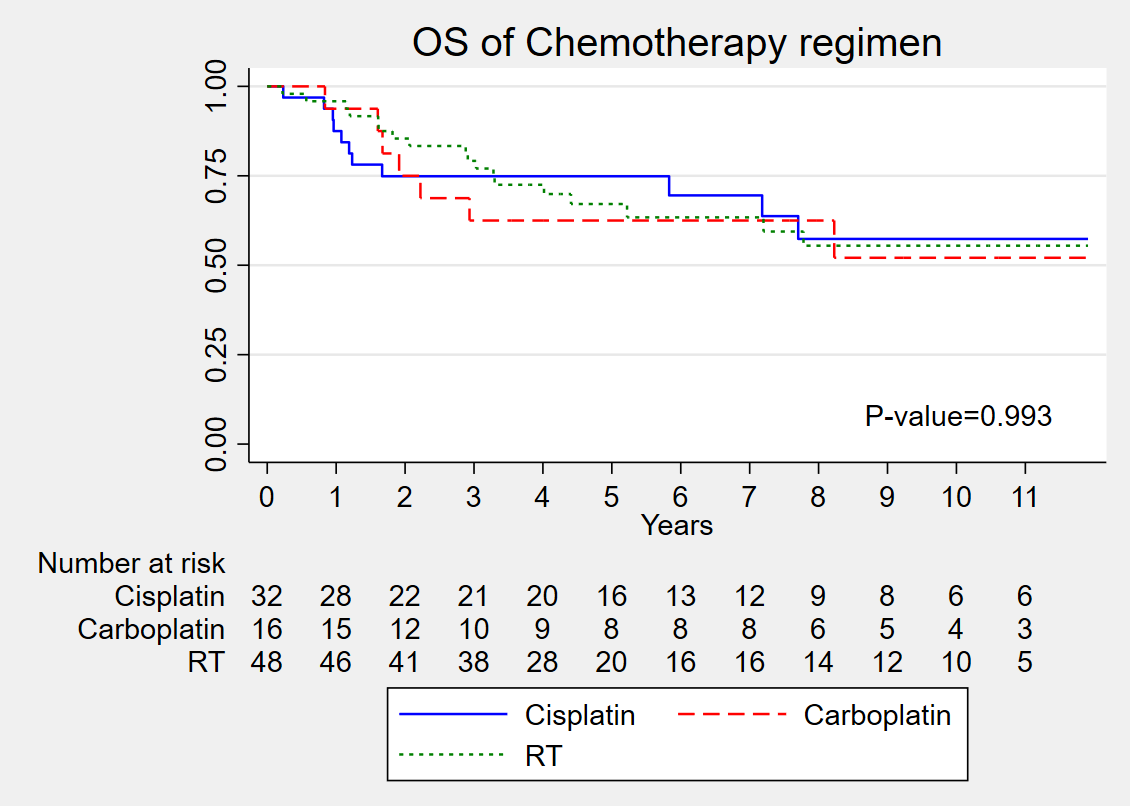


HR 0.98 (95%CI 0.60-1.59); p-value =0.993

Median OS (range) years:

Cisplatin-RT : 5.9 (0.2-15) years

Carboplatin-RT : 6.4 (0.8-15) years

RT alone group : 5.7 (0.2-10) years

**Supplement 13.** EFS by chemotherapy regimen during chemoradiotherapy and radiotherapy alone


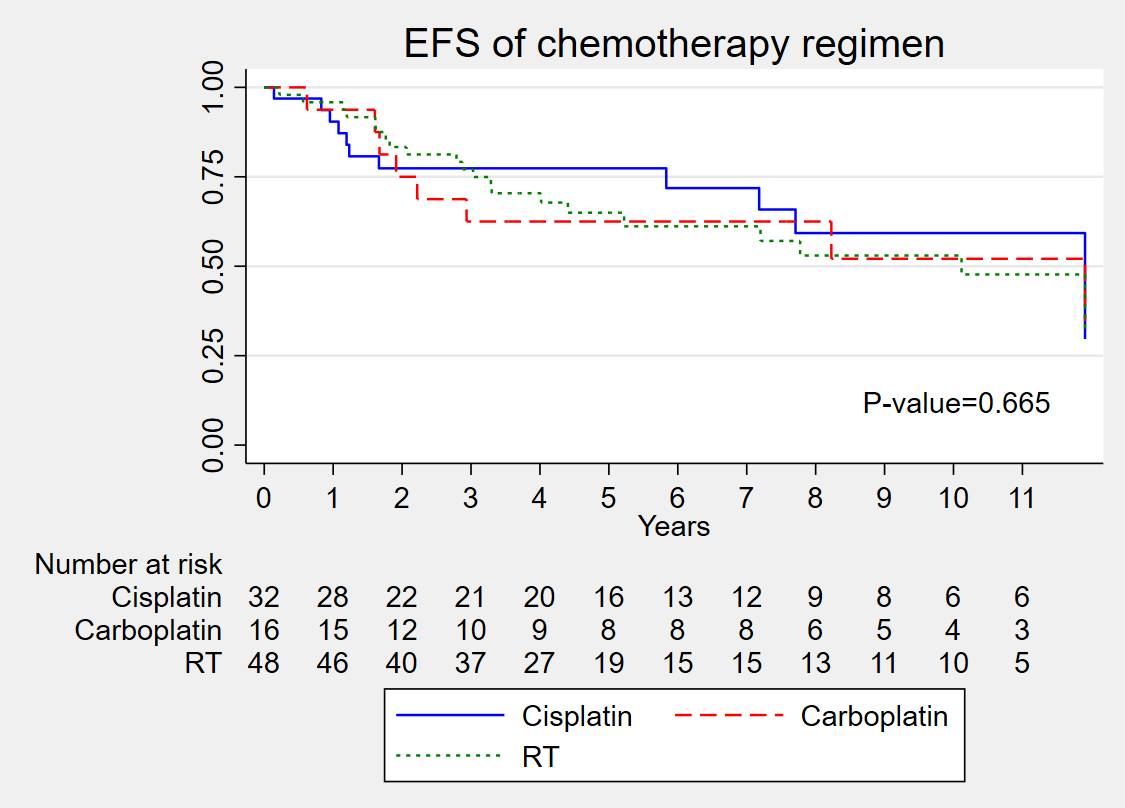


HR 0.94 (95%CI 0.59-1.51); p-value =0.665

Median EFS (range) years:

Cisplatin-RT : 5.8 (0.1-15) years

Carboplatin-RT : 6.4 (0.6-15) years

RT alone group : 5.5 (0.2-10) years
